# Graphene oxide promotes soybean growth by reshaping the rhizosphere microbiome and enhancing soil fertility

**DOI:** 10.3389/fpls.2025.1683882

**Published:** 2025-12-05

**Authors:** Jun Qiao, Lijia Shen, Jiahao Liu, Jiao Sun, Zijun Dai, Jianwen Hu, Changjian Du, JiaSheng Yang, Jingwei Li, Jianguo Zhao, Xiaokang Chen

**Affiliations:** 1 Engineering Research Center of Coal-based Ecological Carbon Sequestration Technology of the Ministry of Education, Shanxi Datong University, Datong, China; 2 Key Laboratory of Graphene Forestry Application of National Forest and Grass Administration, Shanxi Datong University, Datong, China; 3 College of Chemistry and Chemical Engineering, Shanxi Datong University, Datong, China

**Keywords:** graphene oxide, soybean growth, microbial community diversity, sinorhizobium, soil physicochemical properties

## Abstract

Soybean (*Glycine max* L.) one of the world’s most important crops that is prized for its high protein and lipid content. As a prominent member of the carbon nanomaterial family, graphene oxide (GO) exhibits remarkable fertilizer adsorption and slow-release capabilities owing to its high specific surface area and abundant oxygen-containing functional groups, demonstrating broad application prospects in agricultural production. However, its potential role in regulating soybean growth and modulating the rhizosphere microbiome remains poorly understood. To elucidate the mechanism by which GO modulates soybean growth, we investigated eight cultivars (SN24, CD5, 7534, 15GI-16, ZH75, G135, L2012-7, and CD13) with a 30 mg/L GO treatment. The results demonstrate that GO application significantly enhanced key agronomic traits, increasing plant height by 7.17–51.05%, stem diameter by 12.39–63.34%, and the number of root nodules by 33.33–328.57%, along with increase in root biomass. Rhizosphere microbiome analysis revealed that GO restructured microbial communities in L2012–7 and significantly increased bacterial and fungal abundance or diversity based on operational taxonomic unit (OTU) levels (*p* < 0.05). Taxonomic profiling identified GO-enriched beneficial genera (*Sinorhizobium*, *Sphingomonas* and *Trichoderma*), with LEfSe and Random Forest analyses confirming that *Sinorhizobium* is a keystone taxon. Mechanistically, *Sinorhizobium fredii* (*Sf01*) was successfully isolated and identified from soybean rhizosphere soil, which was shown to promote soybean growth. Treatment with 5, 30, and 50 mg/L GO promoted the colony growth of *S. fredii* (*Sf01*) by 40.2%, 42.9%, and 55.5%, respectively, whereas 100 mg/L GO inhibited its growth compared to the control. Furthermore, soil nutrient analysis demonstrated that GO significantly enhanced the contents of soil organic matter, total nitrogen, available potassium, available phosphorus, ammonium nitrogen, and humic acid in soybean rhizosphere soil. Our experimental results demonstrate that GO reshapes the soybean rhizosphere microbial community, which in turn enriches keystone beneficial microbes *S. fredii* (*Sf01*) and enhances soil fertility retention capacity. This cascade of effects collectively promotes soybean plant growth, offering a nano-enabled strategy to reduce reliance on synthetic fertilizers.

## 1 Introduction

With a few decades of rapid development of nanoscience and nanotechnology, carbon nanomaterials (CNMs) have shown promising applications in many fields such as aerospace, biomedicine, and energy (Mbayachi et al., 2021; Jiricková et al., 2022). Simultaneously, they have been considered a promising technology for increasing agricultural production, providing new opportunities and challenges for agricultural innovation (Wu et al., 2023). Graphene oxide (GO), a derivative of graphene, is chemically modified structure with a large number of oxygen-containing hydrophilic functional groups, such as carboxyl, carbonyl, epoxy, and hydroxyl groups (De Jesus et al., 2013). These functional groups endow GO with not only excellent physicochemical properties, including outstanding electrical conductivity and high chemical stability, but also superior surface activity and hydrophilicity, as well as ease of assembly and functionalization. Well-known, GO has been widely applied in agriculture production.

Leguminous plants are one of the most important sources of starch, protein, oil, and vegetables in human foods, having great economic value. Studies have shown that GO can promote the growth and development of leguminous plants (Ge et al., 2024). For instance, Mirza et al. (2022) added different concentrations of GO to the soil of mung beans and their results demonstrated that an appropriate amount of GO positively influences plant growth, increasing root and shoot length, leaf number, root nodules per plant, pod number, and seed number per pod. This study suggested that bioengineered GO could serve as a potential chemical reagent to promote plant growth and significantly increase seed yield per plant. Similarly, graphene oxide enhanced the growth and development of faba beans (Liu et al., 2020; Chen et al., 2024).

*Rhizobium* are a group of Gram-negative bacteria widely distributed in soil that can establish symbiotic nitrogen-fixing relationships with leguminous plants (Pankievicz et al., 2019). They establish a symbiotic nitrogen-fixing system-root nodules-with legumes, converting atmospheric molecular nitrogen into ammonia nitrogen that plants can directly utilize. The symbiotic nitrogen fixation between leguminous plants and rhizobia plays a pivotal role in maintaining the nitrogen cycle of ecosystems and promoting sustainable agricultural development (Yang et al., 2022; Zhang et al., 2022b). Graphene may serve as a carrier for seed and root coatings to enhance rhizobium adsorption. Using hydroponically cultured slow-growing rhizobia and soybeans as experimental subjects, their results demonstrated that graphene promoted the growth of both rhizobia and seeds, serving as a seed carrier for investigating plant-microbe interactions (Sethu Madhavan et al., 2023); a previous report shows that the addition of functionalized graphene altered the relative abundances of nitrogen and phosphorus cycling-related microorganisms in peat soil, thereby modifying the soil’s physicochemical properties, which in turn enhanced the growth of *Vicia faba* plants (Chen et al., 2024). Additionally, the endophytic synthetic bacterial communities entering soybean roots interacted with the plant, increasing the relative abundance of beneficial bacterial genera such as *Nocardioides* and *Pseudonocardia* in the rhizosphere microbial community and enhancing the relative abundance of functional genes related to nitrogen metabolism and indoleacetic acid (IAA) biosynthesis, which enhanced key biological processes such as coenzymes, amino acid transport and metabolism, carbohydrate metabolism and signal transduction were promoted (Wang et al., 2024). Consequently, these changes altered the structure and function of the soybean root bacterial community, effectively promoting soybean plant growth and providing a theoretical basis for the development of microbial fertilizers for soybeans.

Furthermore, synergistic Interaction between GO and rhizobia enhances plant stress tolerance*. Rhizobium* sp. E20–8 alleviates drought stress in maize seedlings through osmotic and antioxidant protection mediated by GO, enhancing plant performance and increasing grain yield under water-deficient conditions (Tiago et al., 2021). Additionally, scientists treated *Artemisia annua* seedlings with GO and found that it promotes their seedings growth. To further investigate the mechanism, an analysis of *A. annua* rhizosphere bacteria revealed that GO (10 and 20 mg/L) increased bacterial diversity and the abundance of carbon cycle-associated communities compared to the control, suggesting that graphene enhances seedling growth by influencing the rhizosphere microbiome (Cao et al., 2024). Lately more studies have shown that 30 mg/L of GO begins to promote an increase in bacterial biomass by the 7^th^ day after treatment, while addition of 20 mg/L of GO enhances the abundance of nitrogen-fixing bacteria by the 7^th^ day of cultivation (Kennedy and Smith, 1995).

The introduction of graphene into soil initiates a cascade of ecological interactions that directly or indirectly reshape soil microbiomes. These interactions induce structural reorganization and functional adaptations within microbial communities (Ren et al., 2015). Crucially, shifts in microbial diversity serve as sensitive biomarkers for evaluating how exogenous materials alter soil metabolic activity and ecological functioning (Insam, 2001). While previous research has shown GO can effectively enhance soybean growth and involves promoting plant growth by enriching beneficial microorganisms (Ge et al., 2024; Wang et al., 2024), this study uses soybeans as the experimental material, systematically comparing the agronomic traits and nodulation responses of eight soybean varieties to GO. To explore the mechanisms, we analyzed the rhizosphere soil microbial community and physicochemical properties in a representative soybean cultivar (L2012-7) that showed the strongest growth promotion under GO treatment. We examined changes in plant growth, rhizobial colonies, and microbial abundance and diversity. This provided insights into GO-soybean-microbe interactions, enabling us to propose a synergistic mechanism for how GO regulates soybean growth. We successfully identified and isolated a key functional microbe, *S. fredii* (*Sf01*). Validation confirmed that graphene directly enhances *Sf01* growth, which then promotes plant development. Field trials demonstrated that graphene application effectively promotes growth across multiple soybean varieties and improves soil fertility. These findings establish a technical basis for precise nanomaterial application in agriculture.

## 2 Materials and methods

### 2.1 Graphene oxide preparation and characterization

This study employed an in-house electrochemical approach to synthesize functionalized GO. In this process, graphite served as both the anode and cathode, with distilled water acting as the electrolyte. By applying a high-frequency pulse current, the graphite electrode underwent electrolysis and oxidation, yielding functionalized graphene. The electrochemical electric field facilitated the intercalation of external electrolyte ions into the graphite layers, mimicking liquid-phase exfoliation. This field-driven process forced electrolyte molecules into the graphite cathode, expanding the interlayer spacing and weakening the van der Waals forces. Ultimately, the electrochemical delamination of graphite−achieved without oxidation−produced the desired functionalized GO. The morphology of GO was characterized by scanning electron microscopy (SEM, TESCAN MAIA 3 LMH) and Raman Spectroscopy (HORIBA, LabRAM HR Evolution) with a 532 nm excitation laser (Renishaw inVia Qontor).

### 2.2 Pretreatment of experiment

#### 2.2.1 Determination of seed germination

The soybean cultivars Lang 2012–7 and ZH75, obtained from Shanxi Agricultural University, were selected, then seeds of the same size were divided into 5 groups:the blank group (CK, refers to treating soybean seeds with sterile water) and four experimental groups labelled as GO10 (10mg/L graphene oxide), GO30 (30mg/L graphene oxide), GO50 (50mg/L graphene oxide) and GO100 (100mg/L graphene oxide); these are first warmed by soaking in water at 55 °C, and then rinsed with sterile water. The seeds were transferred to glass Petri dishes lined with filter paper and treated with different concentrations of GO. Moisture content was maintained at 70% throughout the process. After 48 hours, the germination rate was quantified, and photographic documentation was conducted.

#### 2.2.2 Field planting experiment

Eight soybean cultivars (SN24, CD5, 7534, 15GI-16, ZH75, G135, L2012-7, and CD13) were cultivated at the ‘Changchengshan’ Forest Farm in Datong City, Shanxi Province, China (40.241°N, 113.396°E), a semi-arid temperate continental monsoon climate zone characterized by distinct seasons. The site is located on the Loess Plateau with an average elevation of approximately 1000 meters. The mean annual temperature is 8 °C, and the predominant soil type is cinnamon soil. The experiment was divided into 2 groups, CK and GO30. After sowing, each planting hole was irrigated with 50 mL of the respective GO solution (30 mg/L). This treatment was repeated at 15-day intervals for a total of four applications. After three months, the soybean rhizosphere soil was carefully collected for microbiome analysis. The collected sample was flash-frozen in liquid nitrogen and stored at −80 °C for subsequent microbial sequencing.

### 2.3 Determination of agronomic traits of soybean

The plant heights of eight soybean cultivars were measured using a ruler with a measuring range of 2 m; the distance from the cotyledon node to the apical meristem was defined as plant height. The stem diameter, measured at the middle part above the junction of the plant root and stem, was accomplished with a vernier caliper. The biomass of soybeans was measured using an analytical balance (Shanghai Liang ping, MA200 D1(M)00089). The soybean roots were washed carefully with deionized water, and the root morphology of soybean was determined using an EpsonPerfection V850 Pro (Seiko Epson Corp., Tokyo, Japan) at 600 dpi. The WinRHIZO 4.0 b software was used to analyze the scanned root images.

### 2.4 Microbiome sequencing and analysis

Total DNA was extracted from 0.25 g of soil using the TGuide S96 Magnetic Universal DNA Kit (Tiangen, DP812), with DNA purity and concentration quantified by NanoDrop microspectrophotometry (Thermo Fisher). After dilution to 1 ng/μL with sterile water, the DNA served as the PCR template. For prokaryotic 16S rRNA (V3-V4 region) amplification, primers 338F (5’-ACTCCTACGGGAGGCAGCAG-3’) and 806R (5’-GGACTACHVGGG TWTCTAAT-3’) were employed, while fungal ITS regions were targeted using primers ITS5-F (5’-GGAAGTAAAAGTCGTAACAAGG-3’) and ITS2R (5’-GCTGCGTTCTTCATCG ATGC-3’). PCR reactions (50 μL system) involved initial denaturation at 95 °C for 5 min, 30 cycles of 95 °C/1 min, 60 °C/1 min, and 72 °C/1 min, followed by a final extension at 72 °C for 7 min. Purification with AMPure XP Beads preceded library construction and Illumina novaseq6000 sequencing (Bolyen et al., 2019; Callahan et al., 2016). Then, clean tags were clustered into Operational Taxonomic Units (OTUs) at a 97% similarity threshold using USEARCH (v9.2.64) (Edgar, 2013; Bokulich et al., 2013). Bacterial and fungal OTUs were taxonomically classified with the RDP Classifier (v2.2) against the SILVA (Release 132) and UNITE (Release 8.0) databases, applying a confidence threshold of 0.8 (Wang et al., 2007; Quast et al., 2013; Abarenkov et al., 2010). Community composition was then analyzed across all taxonomic levels (phylum to species).

### 2.5 Diversity analysis and physicochemical property assessment

Inter-group comparisons were performed using Venn diagrams generated with the R VennDiagram package (v1.6.16) (Chen and Boutros, 2011). Alpha diversity indices (Chao1 and Shannon) were computed in QIIME (Caporaso et al., 2010), where: Chao1 estimates species richness and Shannon integrates richness and evenness. Higher values indicate greater diversity. LefSe analysis identified differentially abundant taxa between control and treatment groups across taxonomic levels (Segata et al., 2011). Multivariate analyses including Principal Coordinates Analysis (PCoA), ANOSIM, and Redundancy Analysis (RDA) were conducted using the Vegan package (v2.5.3) in R. Random Forest analysis was performed using QIIME2 with default parameters to differentiate between sample groups (Breiman, 2001; Liaw and Wiener, 2002).

### 2.6 Isolation, purification, and identification of soil microorganisms

According to the method described by Oliveira et al. (2013), three-month-old Lang 2012–7 plants were uprooted from the soil. Soil loosely adhering to the roots was gently shaken into sterile plastic bags, and residual rhizosphere soil was collected using a sterile brush. Subsequently, 10 g of fresh soil was transferred into a conical flask containing 90 mL of sterile water. Under aseptic conditions in a laminar flow hood, the microbial suspension was serially diluted to concentrations of 10^-1^, 10^-2^, 10^-3^, and 10^-4^. Aliquots of each dilution were spread onto beef peptone agar and Potato dextrose agar (PDA) medium (with three replicates per dilution). After incubation at 28 °C for 48 hours, colonies exhibiting distinct morphologies were isolated based on the distribution profiles of predominant rhizosphere microbial genera. These isolates were subsequently subcultured repeatedly until axenic cultures of individual bacterial and fungal strains were obtained. Genomic DNA was extracted from purified microbial isolates using a DNA extraction kit (Tiangen, DP336). PCR amplification was performed using universal primers: 27F (5’-AGTTTGATCMTGGCTCAG-3’) and 1492R (5’-GGTTACCTTGTTACGACTT-3’) for bacterial 16S rRNA genes, and ITS1 (5’-TCCGTAGGTGAACCTGCGG-3′) and ITS4 (5’-TCCTCCGCTTATTGATATGC-3’) for fungal ITS regions. The PCR products were sequenced on an ABI 3730XL platform (Sanger method), and taxonomic identification was achieved by comparing sequences against the NCBI database.

### 2.7 Quantifying graphene’s impact on *Sinorhizobium Fredii*(*Sf01*)colony growth

The colony growth of *Rhizobium* was statistically evaluated after treatment with GO at five different concentrations (0, 5, 25, 50, and 100 mg/L). The bacteria were cultured on AS9 solid medium supplemented with sterilized GO, followed by incubation at 28 °C for 48 h. Post-incubation, colony counts were performed, and growth characteristics (colony size, morphology) were documented photographically for quantitative and qualitative analysis. Data were analyzed using [specify statistical method, e.g., one-way ANOVA] to determine significant differences (*p*< 0.05) in growth inhibition or promotion across GO concentrations.

### 2.8 Determination of physicochemical properties of rhizosphere soil

Soil physicochemical characterization was performed following standardized protocols. For initial measurements, a 10 g aliquot of rhizosphere soil was homogenized in 25 mL deionized water, agitated for 60 seconds, and allowed to settle for 30 minutes prior to pH determination using a calibrated Thermo Orion pH meter (Waltham, MA, USA). Concurrently, bioavailable nutrients including ammonium nitrogen (NH_4_^+^-N), available potassium (AK), and available phosphorus (AP) were quantified using a TPY-8A soil nutrient rapid analyzer (Liu et al., 2020).

Soil organic matter (SOM) content was determined through dichromate oxidation. Briefly, a 1.376 g glucose standard (0.5%-C solution) was prepared in 100 mL volumetric flask with concentrated H_2_SO_4_. Soil samples (1 g) were reacted with 10 mL 0.4 M K_2_Cr_2_O_7_ solution and 10 mL concentrated H_2_SO_4_ under vigorous shaking for 20 minutes. After dilution and settling, supernatant absorbance was measured at 590 nm using a soil nutrient meter. Total nitrogen (TN) was quantified via the Kjeldahl method (HJ 717-2014) (MEP, 2014), while total phosphorus (TP), total potassium (TK) and humic acid (HA) contents were determined according to Chinese agricultural standards NY/T 88-1988, NY/T 87–1988 and GB/T 45891-2025 (Bao, 2005), respectively. All analytical procedures included appropriate quality controls with certified reference materials.

## 3 Results

### 3.1 Characterization of GO

**Figure 1:**
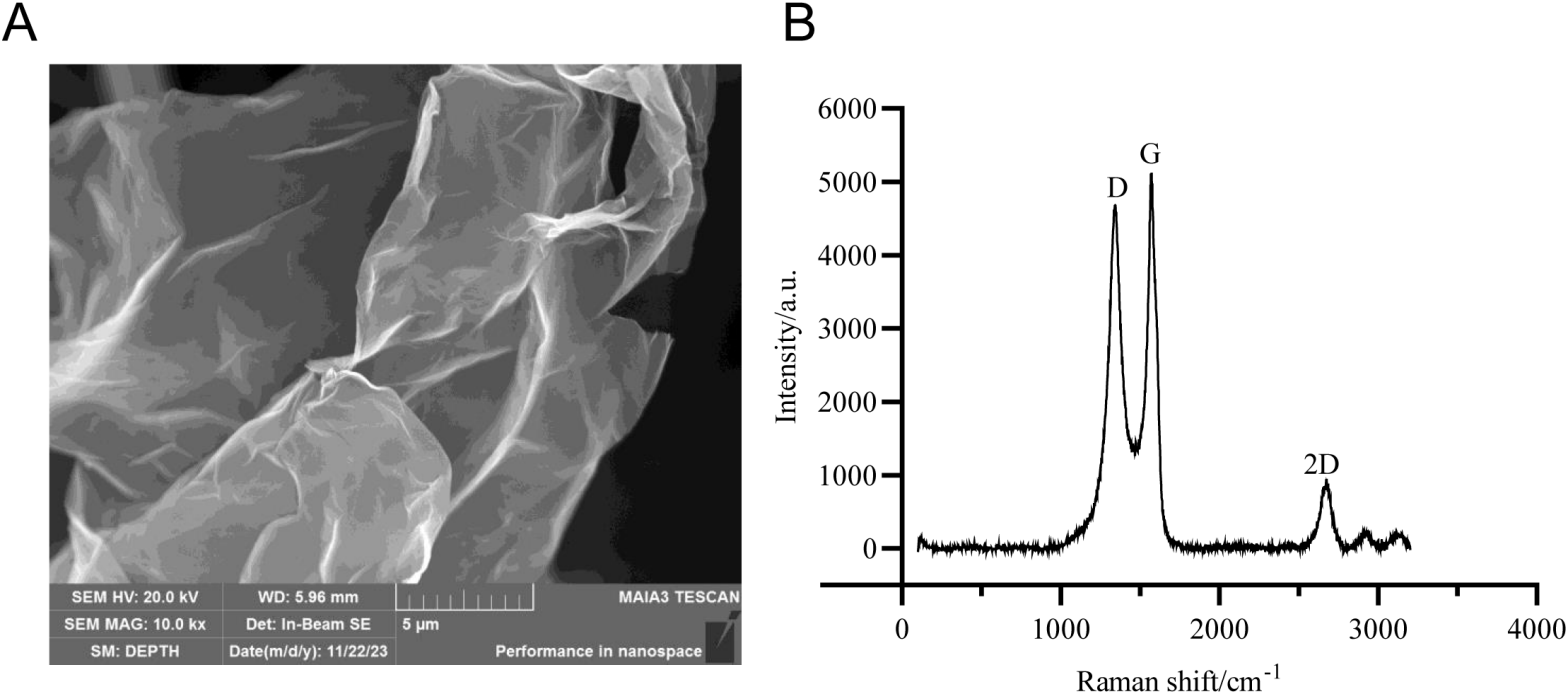
Characterization of GO. **(A)** SEM image; **(B)** Raman spectrum.

The GO was characterized using two analytical techniques. Scanning electron microscopy (SEM) revealed its surface morphology, displaying distinct stacked and folded layers with clearly visible stratification, presenting a crumpled silk-like appearance (**Figure 1A**). **Figure 1B** presents the Raman spectrum of graphene oxide, revealing two characteristic peaks: the G-band and D-band. The G-band, appearing between 1500 cm^-1^ and 1605 cm^-1^, corresponds to the in-plane vibrational mode of sp²-hybridized carbon atoms in the two-dimensional hexagonal lattice. The D-band, observed in the range of 1310–1350 cm^-1^, arises from disordered sp² carbon vibrations.

### 3.2 Application of GO improves the germination rate of soybean seeds

**Figure 2:**
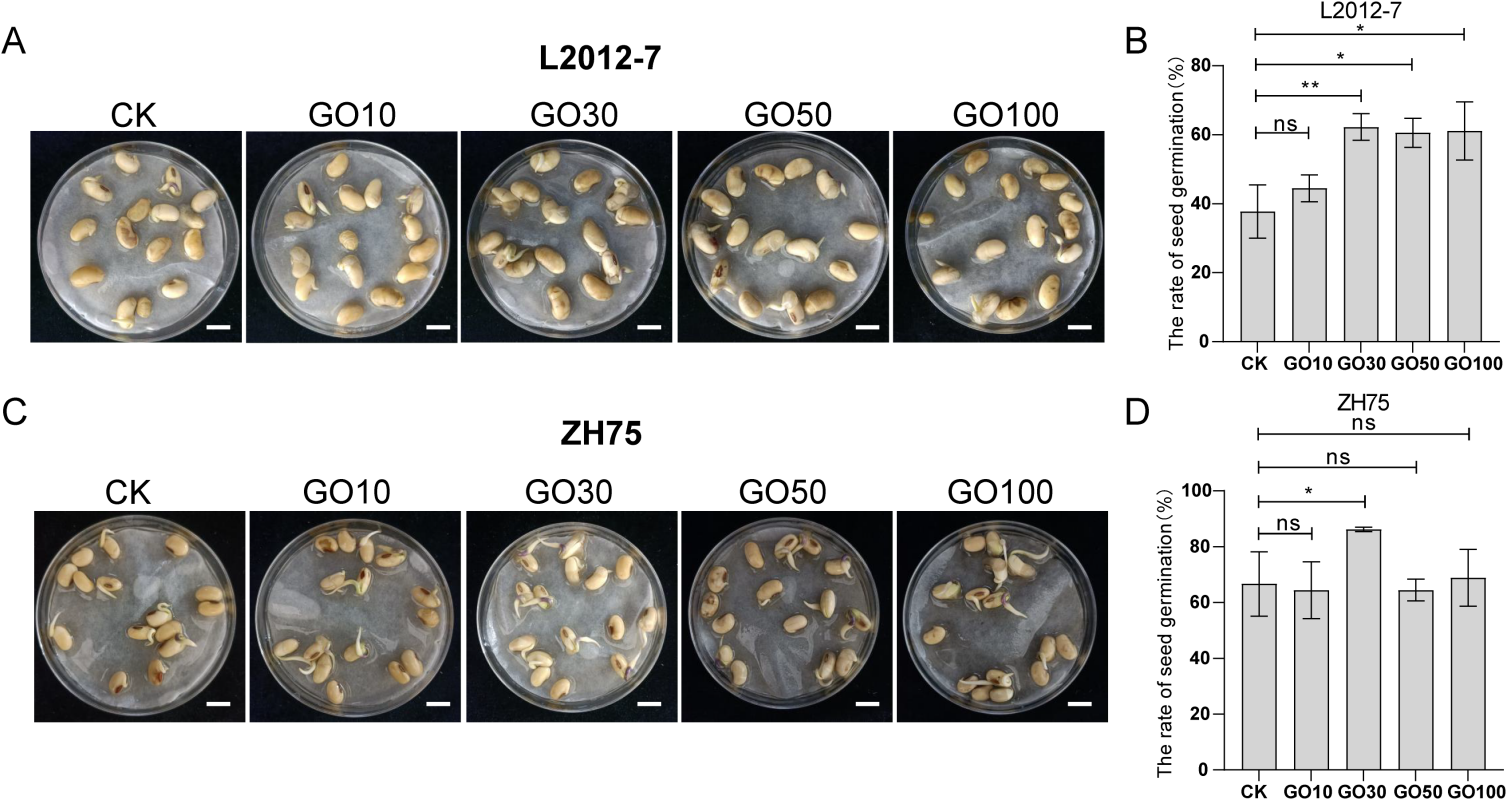
Effects of GO on seed germination in L2012–7 and ZH75 soybean cultivars. **(A)** Representative images of L2012–7 soybean seeds after 48 h GO treatment; **(B)** Bar graph showing germination rates of L2012–7 seeds after 48 h GO treatment; **(C)** Representative images of ZH75 soybean seeds after 48 h GO treatment; **(D)** Bar graph showing germination rates of ZH75 seeds after 48 h GO treatment. Significance is indicated with asterisks (n = 3; one-sided Student’s t-test, *indicates *p*< 0.05, ** indicates *p*< 0.01, ns indicates non-significant differences).

To investigate the effect of GO on the rate of soybean seeds germination, two soybean cultivars, L2012–7 and ZH75, were selected and treated with five concentrations of GO, afterwards their seed germination was evaluated. Our results showed that 10 mg/L GO failed to promote the germination rate in L2012–7 soybean seeds, while concentrations of 30, 50, and 100 mg/L GO significantly enhanced germination compared to the control (**Figures 2A, B**). Additionally, germination experiments with ZH75 soybeans revealed that only 30 mg/L GO significantly improved germination rates relative to the control, without large differences observed at other treatment concentrations (**Figures 2C, D**).

### 3.3 GO treatment promoted plant growth and root development of soybeans

**Figure 3:**
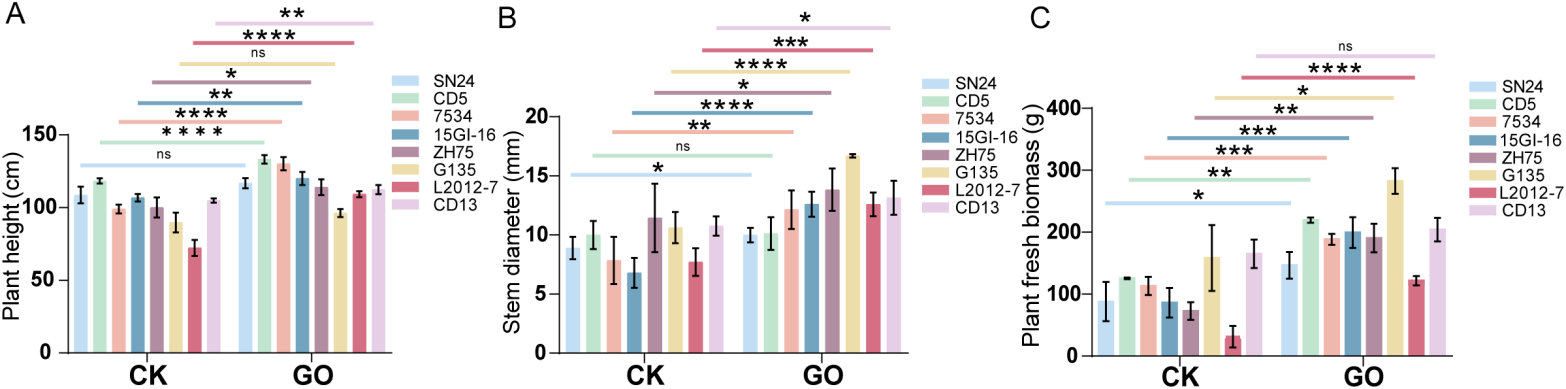
GO promoted the growth of soybean plants. Bar graphs showing the effect of the GO treatment on **(A)** soybean plant height, **(B)** soybean stem diameter, and **(C)** Soybean fresh biomass. Significance is indicated with asterisks (n = 8; one-sided Student’s t-test, *indicates *p*< 0.05, **indicates *p*< 0.01, ***indicates *p*< 0.001, ****indicates *p*< 0.0001, ns indicates non-significant differences).

**Figure 4:**
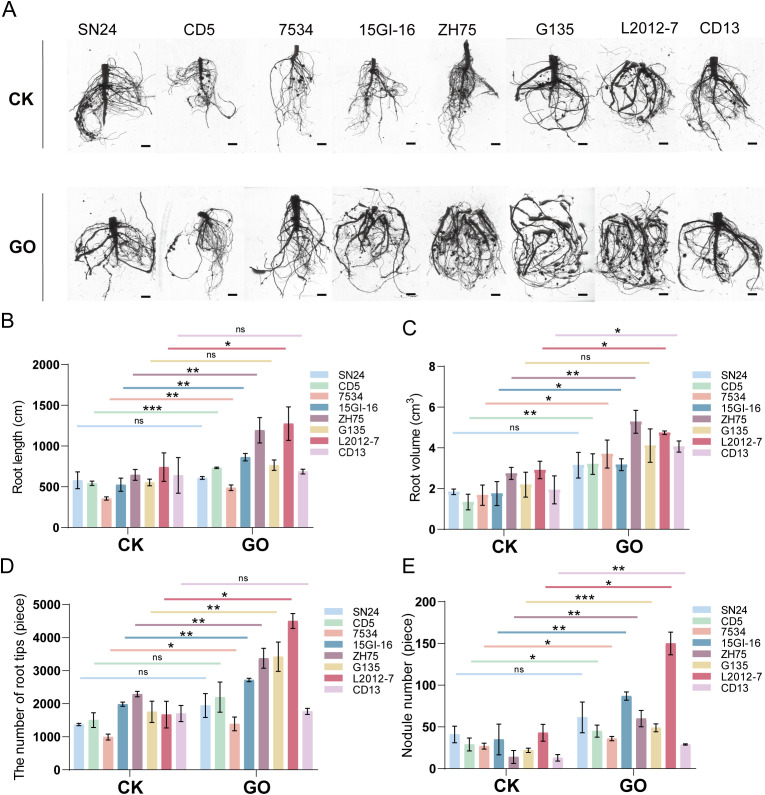
GO promoted the root growth of soybeans plants. **(A)** Root morphology of eight soybean seedlings between Control (CK) and Graphene oxide (GO) groups, scale bar=1cm. Bar graphs showing the impact of the GO treatment on **(B)** soybean root lengths, **(C)** soybean root volumes, **(D)** the number of soybean root tips, and **(E)** the number of soybean nodules. Significance is indicated with asterisks (n = 8; one-sided Student’s t-test, *indicates *p*< 0.05, **indicates *p* < 0.01, ***indicates *p*< 0.001, ns indicates non-significant differences).

We conducted field cultivation experiments using 30 mg/L GO. As shown in **Figure 3**, in comparison to the control, the GO treatment significantly enhances plant height (7.17%-51.05%) (**Figure 3A**; **Supplementary Figure 1**) and stem diameter (12.39%-63.34%; **Figure 3B**) in seven soybean cultivars (SN24, L2012-7, CD13, ZH75, G135, 7534, and 15GI-16), while no significant differences were observed in CD5. Biomass measurements further revealed that the GO treatment significantly increases biomass production in SN24, L2012-7, ZH75, G135, 7534, CD5, and 15GI-16; however no significant effect was detected for CD13 (**Figure 3C**). These results demonstrate that GO significantly promotes growth in most leguminous crops under experimental conditions. As the primary underground organ, plant roots perform crucial functions including water/nutrient absorption, plant anchorage, nutrient storage, and soil ecological interactions. To elucidate the effects of GO on root systems, we examined its impact on soybean roots. **Figure 4** shows that the GO treatment differentially promotes root length (5.11%-85%) (**Figures 4A, B**), root volume (71.12%-120.29%) (**Figure 4C**), and root tip number (3.69%-169.38%) (**Figure 4D**) for all eight soybean cultivars in comparison to the controls. Additionally, we also observed that the GO treatment significantly increases the number of nodules (33.33–328.57%) in seven soybean species except SN24 compared to the control (**Figure 4E**). These results show that GO has a positive effect on the growth of soybeans.

### 3.4 GO altered the microbial community structure in the rhizosphere soil of L2012–7 soybeans

Based on the phenotypic screening results from all eight cultivars, L2012–7 was selected for subsequent microbiome sequencing due to its consistent and significant positive responses to GO application across multiple agronomic traits (**Figures 3**, **4**), making it an ideal model for investigating the mechanisms underlying GO-induced plant growth promotion. The collected samples were subjected to sequencing, with all samples achieving coverage rates above 95%. **Supplementary Figures 2A**, **B** display the rarefaction curves of rhizosphere soil microorganisms in/under different GO concentrations. For bacterial communities, the rarefaction curves plateaued when the sequence numbers reached approximately 58,000 for CK (control) and 60,000 for GO-treated samples. Similarly, for fungal communities, both CK and GO treatments stabilized at around 70,000 sequences, indicating sufficient sequencing depth to reflect the true microbial composition.

**Figure 5:**
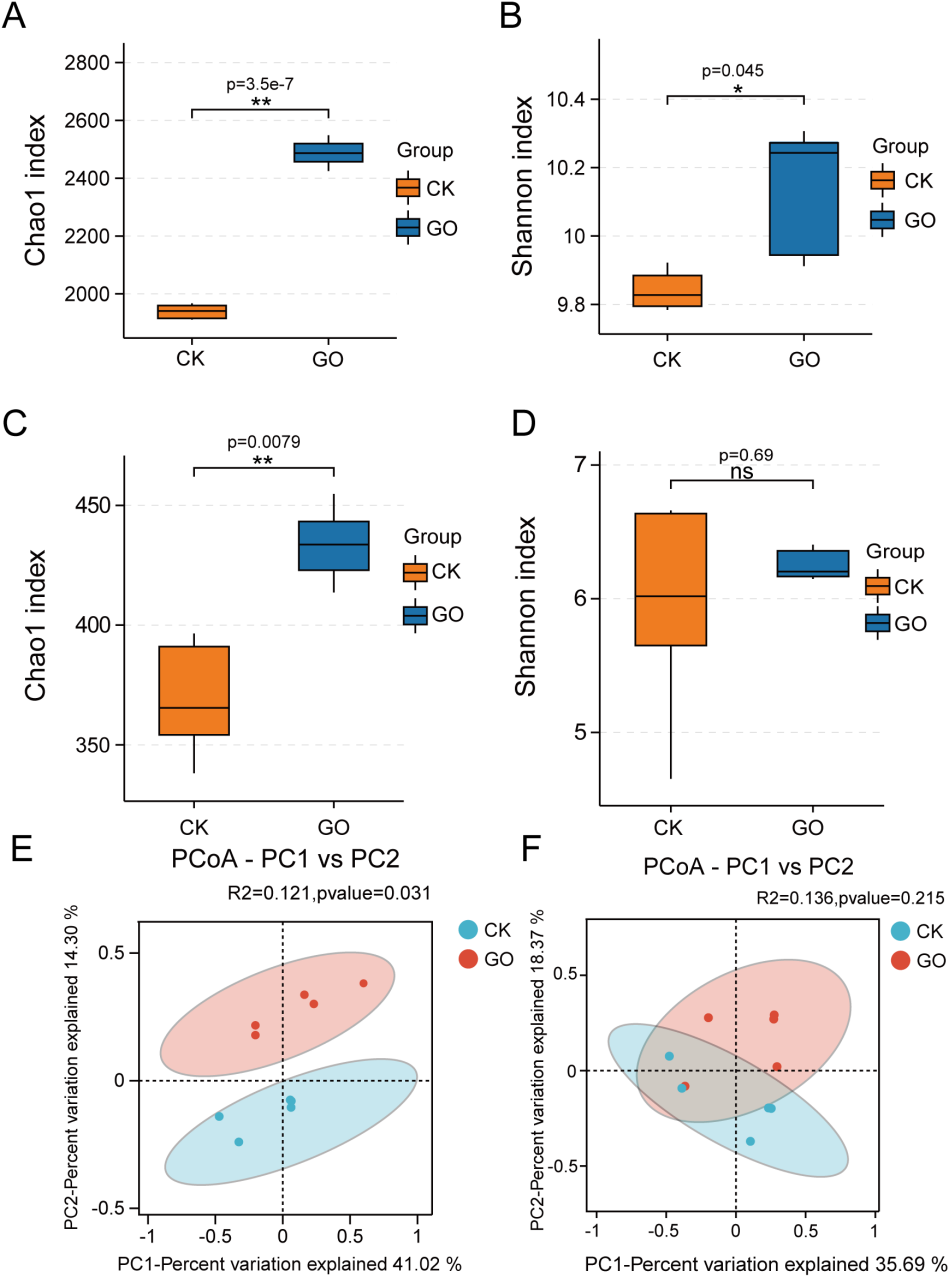
GO alters the abundance and diversity of the soybean rhizosphere microbial community. **(A)** Bacterial Chao1 index in the L2012–7 soybean rhizosphere soil samples with control or GO treatment at the OTU level; **(B)** Bacterial Shannon index in the L2012–7 soybean rhizosphere soil samples with H_2_O or GO treatment at the OTU level; **(C)** Fungal Chao1 index in the L2012–7 soybean rhizosphere soil samples with H_2_O or GO treatment at the OTU level; **(D)** Fungal Shannon index in the L2012–7 soybean rhizosphere soil samples with H_2_O or GO treatment at the OTU level. For **(A-D)**, the significant differences between groups were tested via the Wilcoxon test. The asterisk indicates statistically significant differences between sample treatments at *p* < 0.05 (*), *p* < 0.01 (**), and *p*≥0.05 (ns), “ns” indicates no significant difference between groups. **(E)** PCoA analysis of rhizosphere bacterial β-diversity of soybean plants cultivated in soil; **(F)** PCoA analysis of rhizosphere fungal β-diversity of soybean plants cultivated in soil. Bray–Curtis dissimilarity tests were performed on the taxonomic profile (at the OTU level) for CK and GO microbial.

Cluster analysis was performed, and Venn diagrams were constructed to compare bacterial and fungal OTUs in the soybean rhizosphere. The results revealed a total of 16,758 bacterial OTUs, with 5,970 unique to CK, 9,030 unique to GO, and 1,758 shared between the two treatments, showing a significantly higher bacterial OTU count in GO-treated samples. For fungal OTUs, a total of 2,623 were identified, with 994 unique to CK, 1,226 unique to GO, and 383 shared. These data indicate that the GO treatment significantly increases both bacterial and fungal OTU richness compared to the control (**Supplementary Figures 2C**, **D**). Furthermore, α-diversity analysis at the OTU level shows that GO significantly enhances bacterial diversity, as indicated by higher Chao1 and Shannon indices, as well as fungal richness (Chao1 index) (**Figures 5A–C**), but has no significant effect on the fungal Shannon index (**Figure 5D**). Principal Coordinate Analysis (PCoA) was conducted to assess β-diversity, revealing significant differences in bacterial communities between CK and GO (Bray-Curtis, PERMANOVA, *p* < 0.05), while no significant difference was presented in fungal communities (*p*>0.05) (**Figures 5E, F**).

### 3.5 GO recruits potentially beneficial microbes in the soybean rhizosphere, including *Sinorhizobium*, *Sphingomonas*, and *Mortierella*

**Figure 6:**
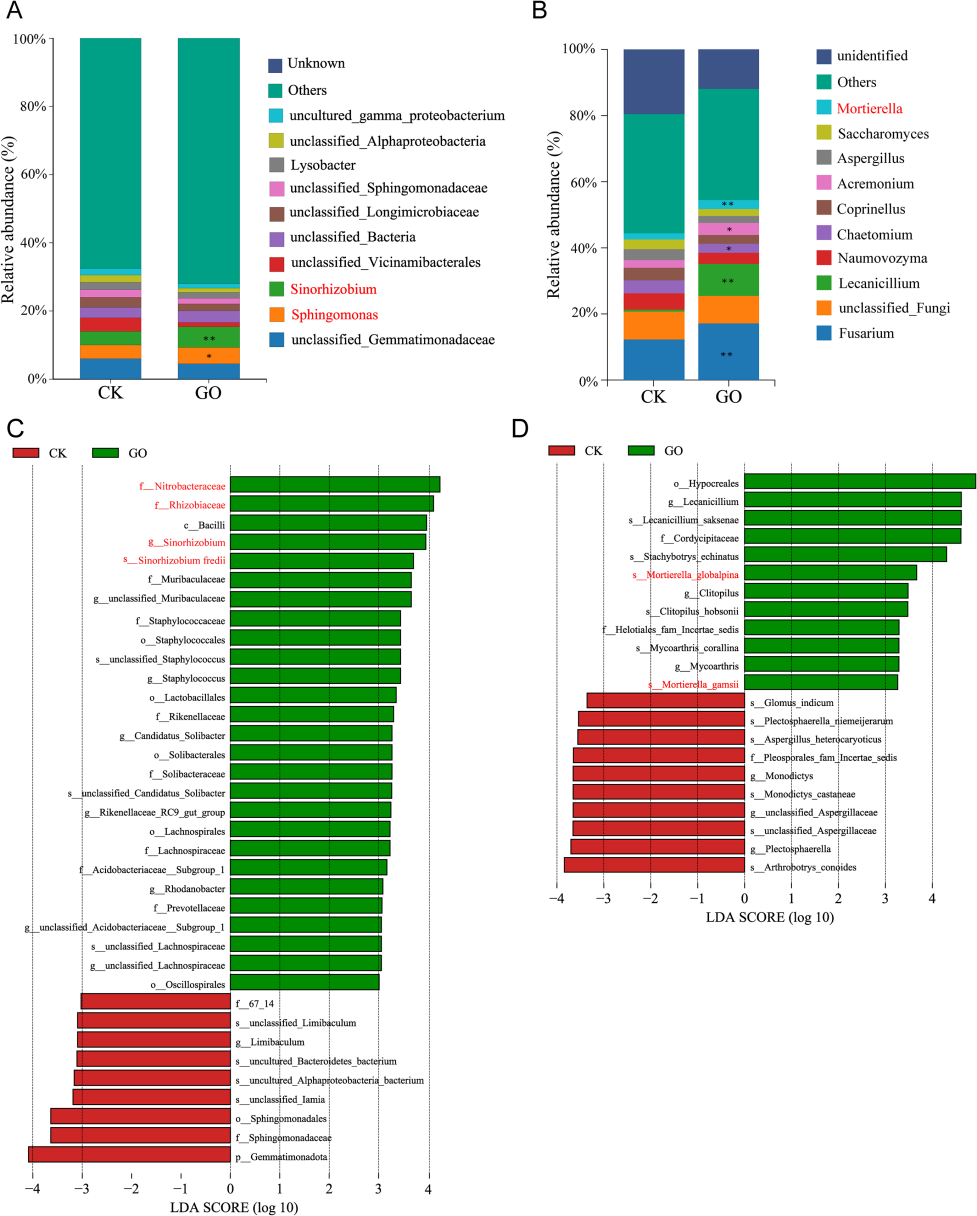
Differentially enriched species in the Graphene oxide (GO) and Control (CK) groups. Relative abundance of **(A)** major bacteria and **(B)** major fungi, both at genus levels (%). **(C)** Bacterial and **(D)** fungal LEfSe for GO compared with the CK group. Potentially beneficial microorganisms in the figure are labeled in red.

The analysis of the relative abundance of bacteria and fungi at the genus level in soybean rhizosphere soil samples revealed that the top 10 bacterial genera in both sample groups were *uncultured_gamma_proteobacterium*, *unclassified Alphaproteobacteria*, *Lysobacter*, *unclassified Sphingomonadaceae*, *unclassified _Longimicrobiaceae*, *unclassified Bacteria*, *unclassified Vicinamibacterales*, *Sinorhizobium*, *Sphingomonas*, and *unclassified Gemmatimonadaceae*. The study showed that, compared to the control, GO significantly increased the abundance of *Sinorhizobium* and *Sphingomonas* (**Figure 6A**). The top 10 fungal genera in relative abundance were *Mortierella*, *Saccharomyces*, *Aspergillus*, *Acremonium*, *Coprinellus*, *Chaetomium*, *Naumovozyma*, *Lecanicillium*, *unclassified_Fungi*, and *Fusarium*. Among these, the relative abundances of *Mortierella*, *Acremonium*, *Lecanicillium*, and *Fusarium* were significantly higher than those in the control group (**Figure 6B**).

The Linear Discriminant Analysis Effect Size (LEfSe) results for microorganisms revealed that, compared to the control group, the GO treatment of the rhizosphere soil of Lang 2012–7 soybeans significantly increased the abundance of the top five biomarker microbes: *f_Nitrobacteraceae*, *f_Rhizobiaceae*, *c_Bacilli*, *g_Sinorhizobium*, and *s_Sinorhizobium fredii*; these play crucial roles in plant-microbe interactions. Notably, the abundance of *g_Sinorhizobium* and *s_Sinorhizobium fredii* was significantly higher than that in the control group, suggesting their important role in responding to GO-promoted growth of Lang 2012–7 soybean plants (**Figure 6C**). The fungal LEfSe results showed that, compared to the control, GO significantly enhanced the abundance of *o_Hypocreales*, *g_Lecanicillium*, *s_Lecanicillium saksenae*, *f_Cordycipitaceae*, and *s_Stachybotrys echinatus*. Additionally, GO promotes the abundance of several *Mortierella* species (*s_Mortierella_globalpina* and *s_Mortierella_gamsii*) (**Figure 6D**); previous research indicates that *Mortierella* can prevent soil degradation, improve the state of soil health, and promote crop growth (Li et al., 2018). Previous research has shown that *Sphingomonas*, as a type of plant growth-promoting rhizobacteria (PGPR), significantly enhances the growth and nutritional content of spinach (Sultana et al., 2024). These findings indicate that GO induces an increase in the abundance of potentially beneficial microbes in the soybean rhizosphere, including rhizobia *Sinorhizobium*, *Sphingomonas*, and *Mortierella*.

### 3.6 GO promotes the growth of *Sinorhizobium fredii*(*Sf01*)

To further validate the effects of GO on soil microorganisms, we isolated, screened, and identified microbes from the rhizosphere soil of Lang 2012–7 soybeans (**Supplementary Figures 3**, **4**). Ultimately, we identified the rhizobial strain *Sinorhizobium fredii* (Sf01), and subsequent inoculation experiments demonstrated its ability to promote soybean growth (**Supplementary Figure 5**).

**Figure 7:**
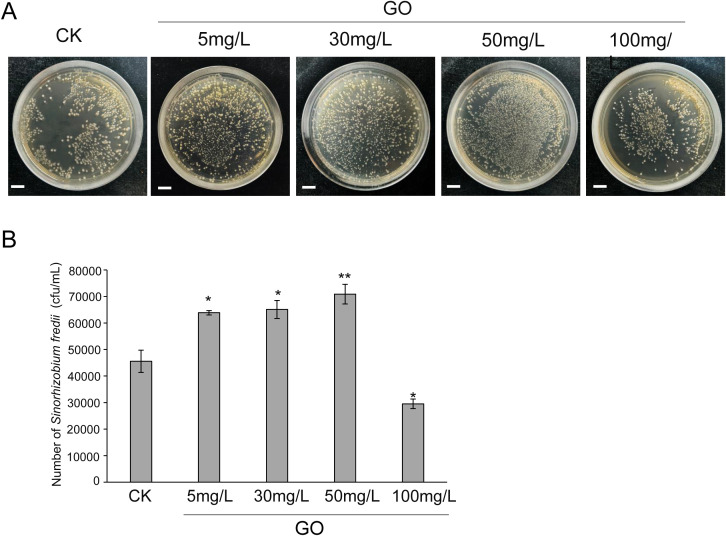
Effect of GO on the growth of *Sinorhizobium fredii*(*Sf01*). **(A)** Representative images illustrate the growth of rhizobia after 48-hour GO treatment; **(B)** Bar graph show the quantitative analysis of rhizobial colony counts from plate cultures, Significance is indicated with asterisks (n = 3; one-sided Student’s t-test, *indicates *p*< 0.05, **indicates *p* < 0.01).

To further elucidate the impact of GO on soybean rhizobia, we treated the *Sf01* strains with different concentrations of GO. The results revealed that, compared to the control, GO at concentrations of 5, 30 and 50 mg/L significantly enhances *Sf01* growth, with increased rates of 40.2%, 42.9%, and 55.5%, respectively. In contrast, 100 mg/L GO inhibited *Sf01* growth, reducing it by 35.1% (**Figure 7**).

### 3.7 GO increased the nutrient content in soybean rhizosphere soil

**Figure 8:**
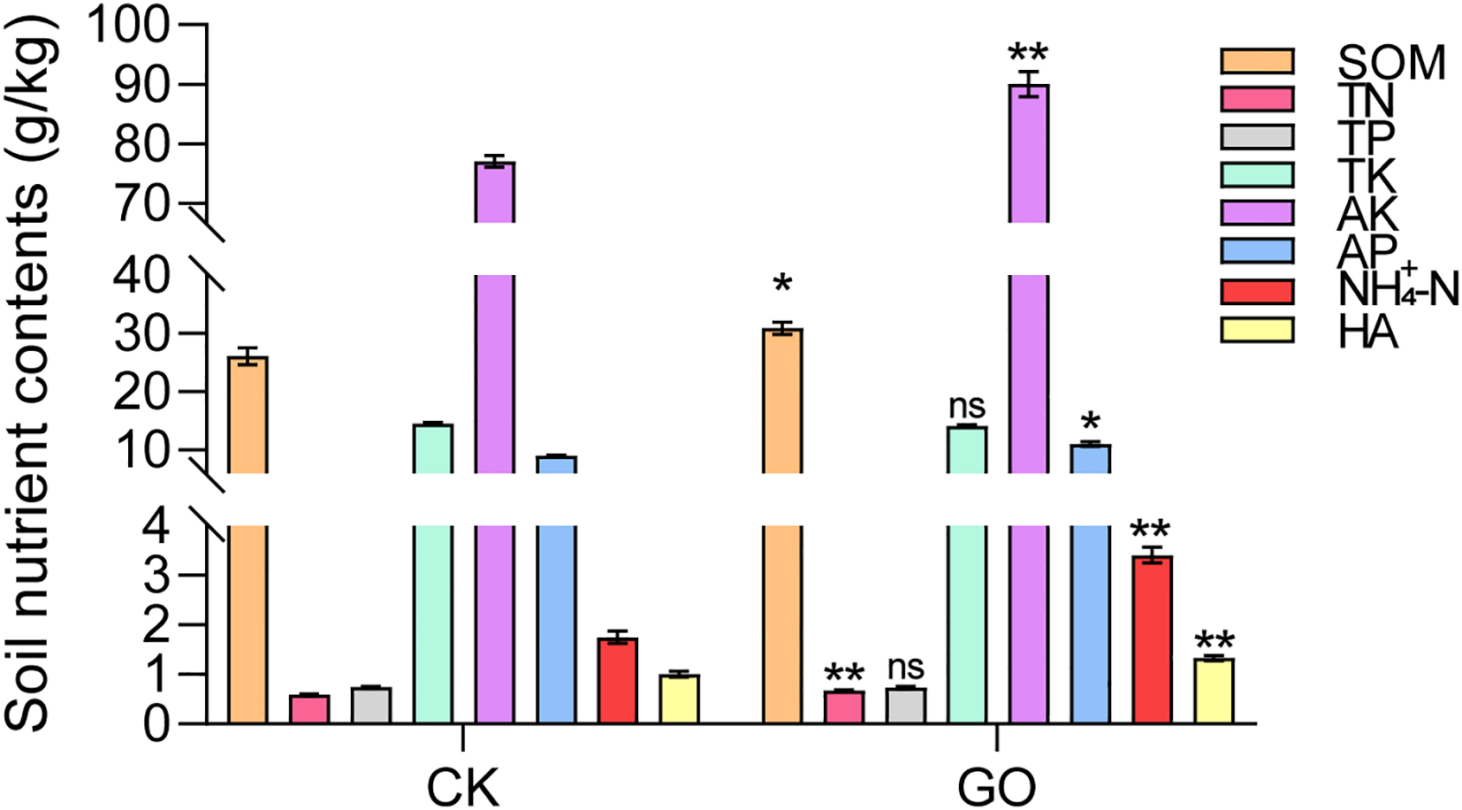
GO improved the physicochemical properties of soybean rhizosphere soil. Significance is indicated with asterisks (n = 3; one-sided Student’s t-test, *indicates *p*< 0.05, **indicates *p*< 0.01, ns indicates non-significant differences).

Physicochemical analysis of the rhizosphere soil of L2012–7 soybeans revealed that, compared to the control, GO significantly increased the content of SOM (soil organic matter), TN, AK, AP, NH_4_^+^-N (ammonium nitrogen), and HA (humic acid). However, no significant changes were observed in the levels of TP (total phosphorus) and TK (**Figure 8**). Furthermore, correlation analysis between core soil microorganisms and soil nutrients under graphene treatment revealed that *Sinorhizobium* and *Sphingomonas* were positively correlated with SOM, TN, and AK, but negatively correlated with TP. Notably, *Sinorhizobium* showed statistically significant correlations with carbon- and nitrogen-related nutrient levels (**Supplementary Figure 6**). These results demonstrate that the GO-induced increase in soil fertility is associated with the enrichment of certain keystone microbes.

## 4 Discussion

GO is considered to have great potential in agricultural applications due to its abundant oxygen-containing functional groups, large specific surface area, and high conductivity (Ahmad et al., 2018). Previous studies have shown that different concentrations of graphene derivatives exert different effects on the growth and development of plant radicles. For instance, 200 mg/L and 1000 mg/L GO significantly enhanced the germination rates of tomato and pepper seeds, respectively (Ge et al., 2022). Additionally, graphene has been found to markedly promote the germination and root growth of mung beans (Zhang et al., 2022a). In our study, we observed that GO at concentrations of 30 mg/L, 50 mg/L, and 100 mg/L, all facilitates seed germination in soybean cultivars Lang 2012–7 and ZH 75 (**Figure 2**). All these studies suggest that graphene-based materials can enhance seed germination to diverse plant species.

Plant roots play a crucial role in growth and environmental adaptation, performing essential physiological functions such as nutrient uptake, anchorage, storage, and signal transduction. Further studies have shown that low concentrations of graphene can effectively promote root development. For example, GO at 20 mg/L and 30 mg/L significantly enhances root growth in tobacco and lettuce, respectively, increasing adventitious root number and fresh root weight (Gao et al., 2020; Jiao et al., 2016). Similarly, graphene treatment significantly improves root biomass, total root length, surface area, and volume in *Aloe vera* (Zhang et al., 2021).

A study of 48 plant species revealed that 25 mg/L graphene treatment increases total root length in 69.77% of the plants, inhibiting growth in only 11.63%, and has no significant effect on the remaining 18.6%, further confirming its positive regulatory role in root systems (Chen et al., 2022b). Additionally, GO differentially influences functional genes related to root growth. For instance, it upregulates the expression of IQM3, ARF7, and ARF19 in Arabidopsis, promoting primary root elongation and lateral root formation (Gao et al., 2022). Graphene can also enhance artemisinin production in Artemisia annua by modulating the miR828-AaMYB17 pathway to stimulate trichome development (Cao et al., 2024).

In this study, we evaluated the effects of 30 mg/L GO on eight leguminous plants. The results demonstrated that GO significantly improved agronomic traits, including plant height, stem diameter, root growth, and biomass, in most species (**Figures 3**, **4**), further demonstrating its beneficial biological effects.

Graphene nanomaterials can optimize the rhizosphere microenvironment and enhance the absorption efficiency of water and nutrients, thereby promoting root growth. Graphene also improves the uptake efficiency of mineral elements (e.g., nitrogen, phosphorus, potassium) by plant roots. Possible mechanisms by which GO influences the plant rhizosphere microbial community structure may include the following: on the one hand, as shown in **Figure 8**, GO possesses a large specific surface area and strong adsorption capacity, which enhance the soil’s ability to retain nutrients such as nitrogen, phosphorus, and potassium. This provides an abundant substrate of nutrients for the growth and reproduction of microorganisms. The enrichment of nutrients directly stimulates microbial metabolic activity, increases microbial biomass, and attracts microbial taxa capable of efficiently utilizing these nutrients to accumulate in the area, thereby altering the community structure (Chen et al., 2021, 2024; Huo et al., 2025). On the other hand, the various oxygen-containing functional groups on the surface of GO can effectively retain moisture in the rhizosphere soil, improving soil water conditions around plant roots and creating a favorable environment for plant growth (Yin et al., 2018; Zhao et al., 2022). Moreover, GO can engage in direct interactions with microorganisms. These microbes can utilize GO as a carbon source, supporting their metabolic processes and further enhancing their growth (Qu et al., 2018). In this study, we found that GO significantly increased the contents of SOM, TN, AP, NH_4_^+^-N, and HA in soybean rhizosphere soil (**Figure 8**). Humic acid is a natural organic compound widely present in soil, known for its multiple functions such as improving soil structure and promoting plant growth (Galambos et al., 2020). In this study, it was observed that graphene promoted the accumulation of humic acid in soil, which may be attributed to the large specific surface area and abundant π–π conjugated structures of graphene oxide. These properties enable it to strongly adsorb organic molecules in soil or culture media through π–π interactions, hydrophobic effects, and van der Waals forces. Such adsorption enriches these precursor substances on the surface of graphene, increasing their local concentration and thereby facilitating the formation of humic acid. Furthermore, graphene nanomaterials can form stable complexes with trace elements, thereby enhancing the transport efficiency of nutrients (Yin et al., 2018). Additionally, the oxygen-containing functional groups on the graphene surface effectively enrich soil moisture and nutrients in the rhizosphere, inducing the upregulation of genes encoding potassium and ammonium ion transporters (Chen et al., 2022a, 2021). Notably, GO treatment led to a significant enrichment of beneficial bacteria, including *Sinorhizobium*, *Sphingomonas*, and *Mortierella*. This finding suggests that GO may directly interact with the cell surface characteristics or metabolic pathways of these specific taxa (**Figure 6**). These mechanisms promote root and plant growth, shorten the seedling cultivation period, and improve the quality of seedling propagation.

Soil microorganisms, characterized by their vast diversity and abundance, constitute a natural organic component of soil and play a crucial role in the transformation of organic matter, soil improvement, and ecological restoration. The persistent presence of graphene in soil can alter the composition of microbial communities, thereby influencing microbial mineralization, nitrogen fixation, and plant growth promotion (Kennedy and Smith, 1995). Studies have demonstrated that graphene modifies the rhizosphere microbial community structure, subsequently enhancing plant growth and quality (Chen et al., 2024; Cao et al., 2024; You et al., 2023). In this study, we examined the impact of GO on the rhizosphere microbiota of soybean cultivar ‘Lang 2012-7’. The results revealed that GO significantly increases the abundance and diversity of rhizosphere microbial communities compared to the control (**Figure 5**). Differential species analysis indicates that GO induced an increase in the abundance of potentially beneficial microorganisms, including *Rhizobium*, *Sphingomonas*, and *Trichoderma* (**Figure 6**). Notably, *Sinorhizobium fredii* (*Sf01*) was identified in the rhizosphere soil (**Supplementary Figures 3**, **4**). Further functional validation confirmed that GO at concentrations of 5, 30 and 50 mg/L significantly promotes *Sf01* colony growth (**Figure 7**), aligning with our field observations that GO increases soybean nodulation (**Figure 4E**). However, high concentrations of GO (100 mg/L) inhibits *Rhizobium* growth, likely due to dose-dependent effects and graphene’s physical-chemical properties. For instance, the sharp edges of graphene can puncture cell membranes, leading to cytoplasmic leakage (physical piercing) (Al-Jumaili et al., 2017; Chandraker et al., 2017). Additionally, GO can induce reactive oxygen species (ROS) generation, triggering oxidative stress and cellular inactivation (chemical oxidation) (Ahmed and Rodrigues, 2013). Moreover, GO can envelop the cells, forming a physical barrier that impedes nutrient uptake and suppresses cellular respiration, ultimately inhibiting bacterial growth (Akhavan et al., 2011).

Collectively, these findings suggest that GO modulates rhizosphere microbial community structure, thereby influencing plant growth. Regarding graphene’s transformation mechanisms in soil, prior research has identified microorganisms capable of utilizing graphene as a sole carbon source for growth and degradation (Qu et al., 2018). Furthermore, a study by Mao’s research group at Nanjing University employed ^14^C-labeled graphene to investigate its uptake, distribution, translocation, and transformation in rice. The results demonstrated that graphene could penetrate cell walls and membranes, accumulating in chloroplasts, while hydroxyl radicals were detected in graphene-exposed leaves. The captured ^14^CO_2_ confirmed that graphene in rice stems and leaves undergoes mineralization (Huang et al., 2018). These results indicate that graphene can be effectively degraded and metabolized by soil microorganisms. However, the specific mechanisms of microbial degradation, transformation, and plant assimilation of graphene in soil must be further elucidated.

## 5 Conclusions

**Figure 9:**
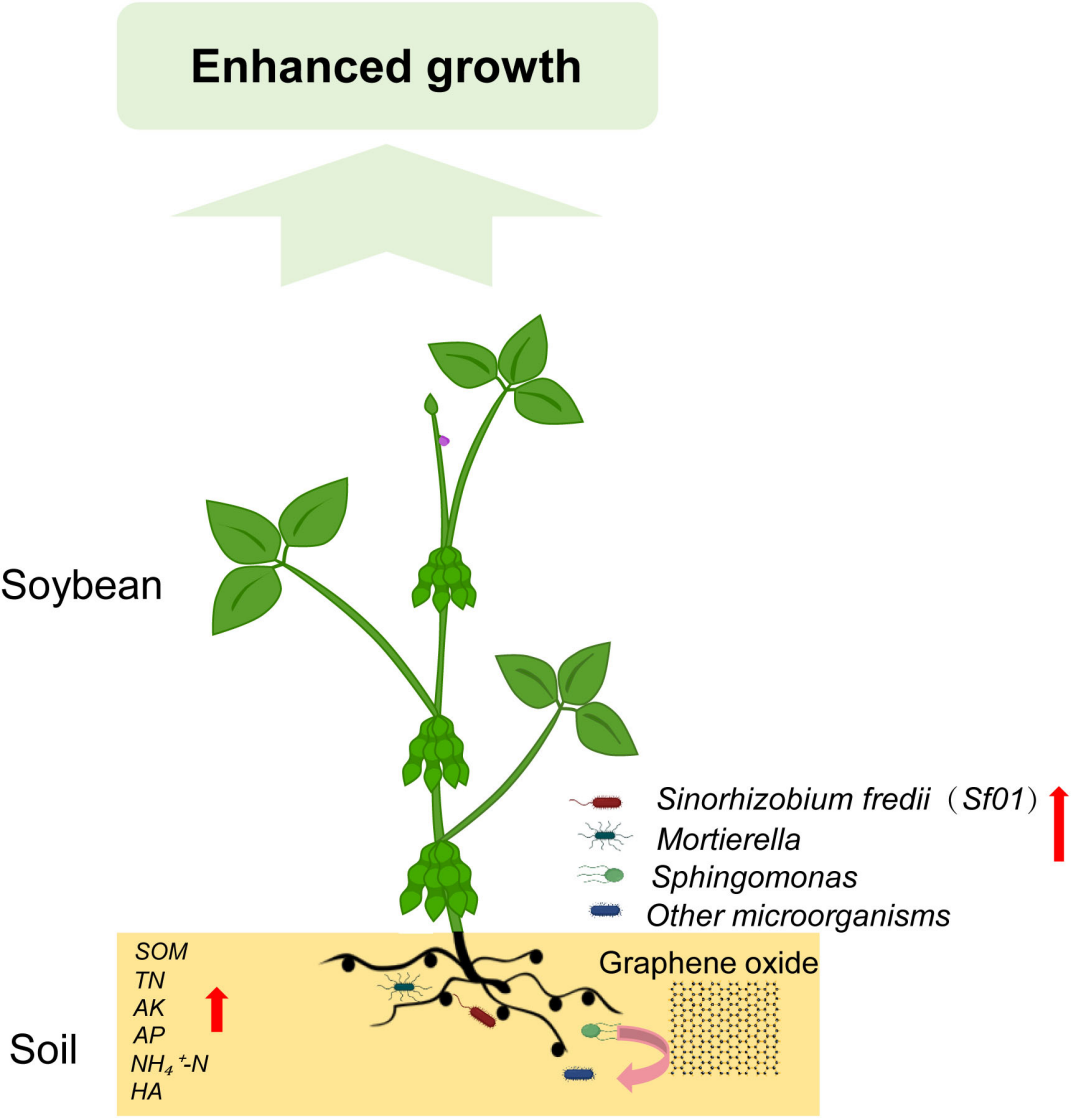
Proposed model for GO promoting soybean growth. GO enhances soybean growth by modulating the diversity of the rhizosphere microbial community, enriching beneficial microorganisms such as *Sinorhizobium fredii(Sf01)*, *Sphingomonas*, and *Mortierella*, while simultaneously improving soil fertility.

Seed treatment with GO at 30 mg/L was identified as optimal for enhancing germination in soybean cultivars ‘Lang 2012-7’ and ‘Zhonghuang 75’. Field trials evaluating its impact on eight legume species demonstrated that GO significantly improves key agronomic traits, including plant height, stem diameter, root growth, and biomass accumulation in most species. Microbiome analysis revealed that GO enriches rhizosphere microbial diversity and abundance, particularly stimulating the proliferation of beneficial taxa such as *Sinorhizobium*, *Sphingomonas*, and *Trichoderma*, which contribute to enhanced plant growth. Isolation and identification of rhizosphere microbiota led to the discovery of *Sinorhizobium fredii* in GO-treated soils. Subsequent experiments showed that 5, 30 and 50 mg/L GO markedly promotes *Sinorhizobium fredii* (*Sf01*) colony growth, whereas 100 mg/L GO exerted inhibitory effects. Additional soil physicochemical analyses indicated that GO markedly increases the content of soil organic matter, total nitrogen, available phosphorus, readily available potassium, ammonium nitrogen, and humic acid, suggesting its role in improving soil quality (**Figure 9**). These findings offer technical support for creating “GO-rhizobia” composite inoculants at an optimal concentration to promote plant growth, enhance biological nitrogen fixation, and improve soil fertility, thereby advancing sustainable agriculture.

## Data Availability

The Microbiome data presented in the study are deposited in the Sequence Read Archive database of NCBI repository, accession number PRJNA1367008.
