# Supplementary material for: Graphene oxide promotes soybean growth by reshaping the rhizosphere microbiome and enhancing soil fertility
Source: Front Plant Sci. 2025 Dec 5;16:1683882. doi: 10.3389/fpls.2025.1683882 (PMC12714940; doi:10.3389/fpls.2025.1683882)
Supplement: Supplementary file 1 [file Supplementaryfile1.docx]

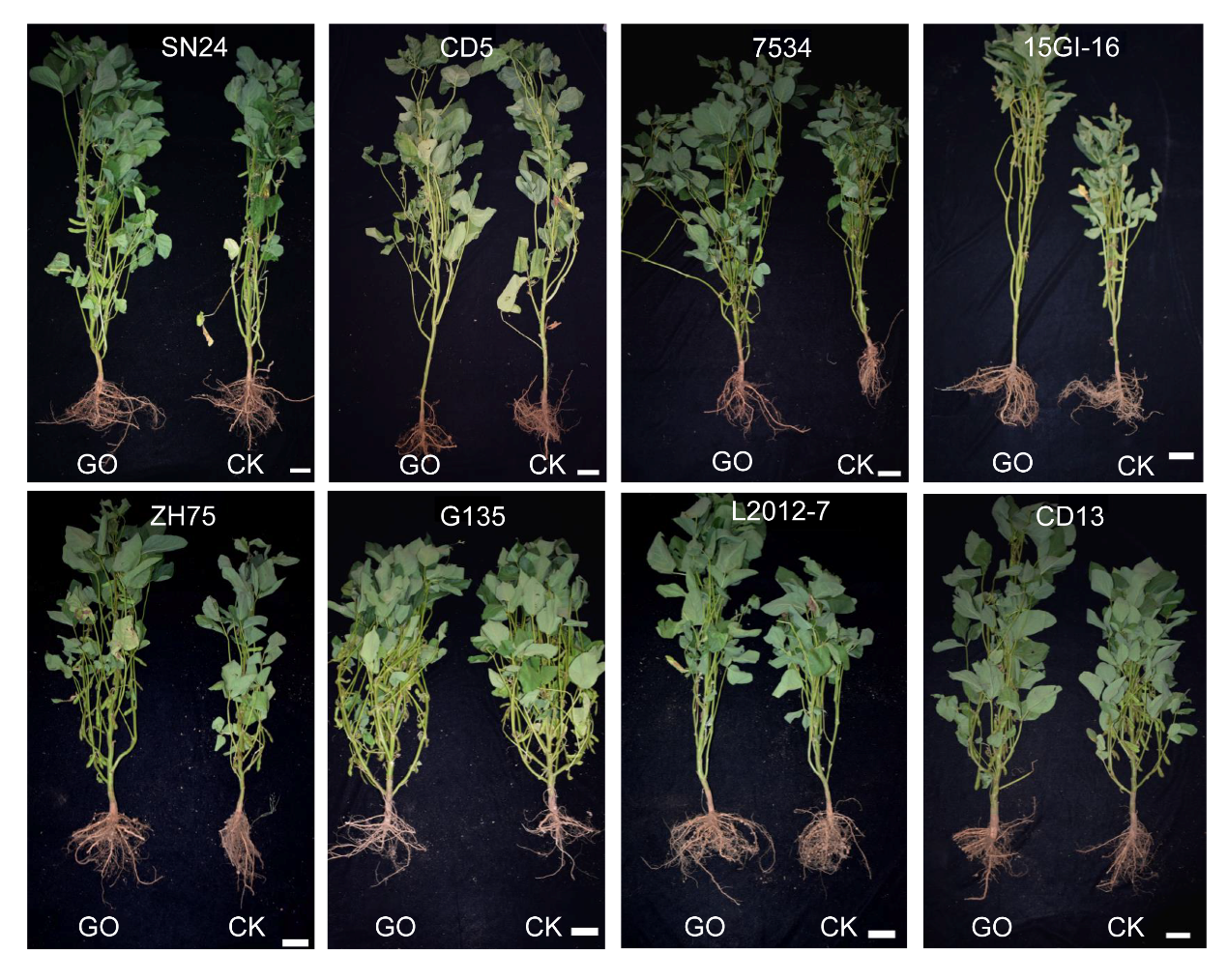


**Supplementary Figure 1.** Representative images of growth phenotypes of soyabean 8 cultivars plants after a GO treatment. Representative images were taken three months after treated with GO, the scale bar is 1 cm.


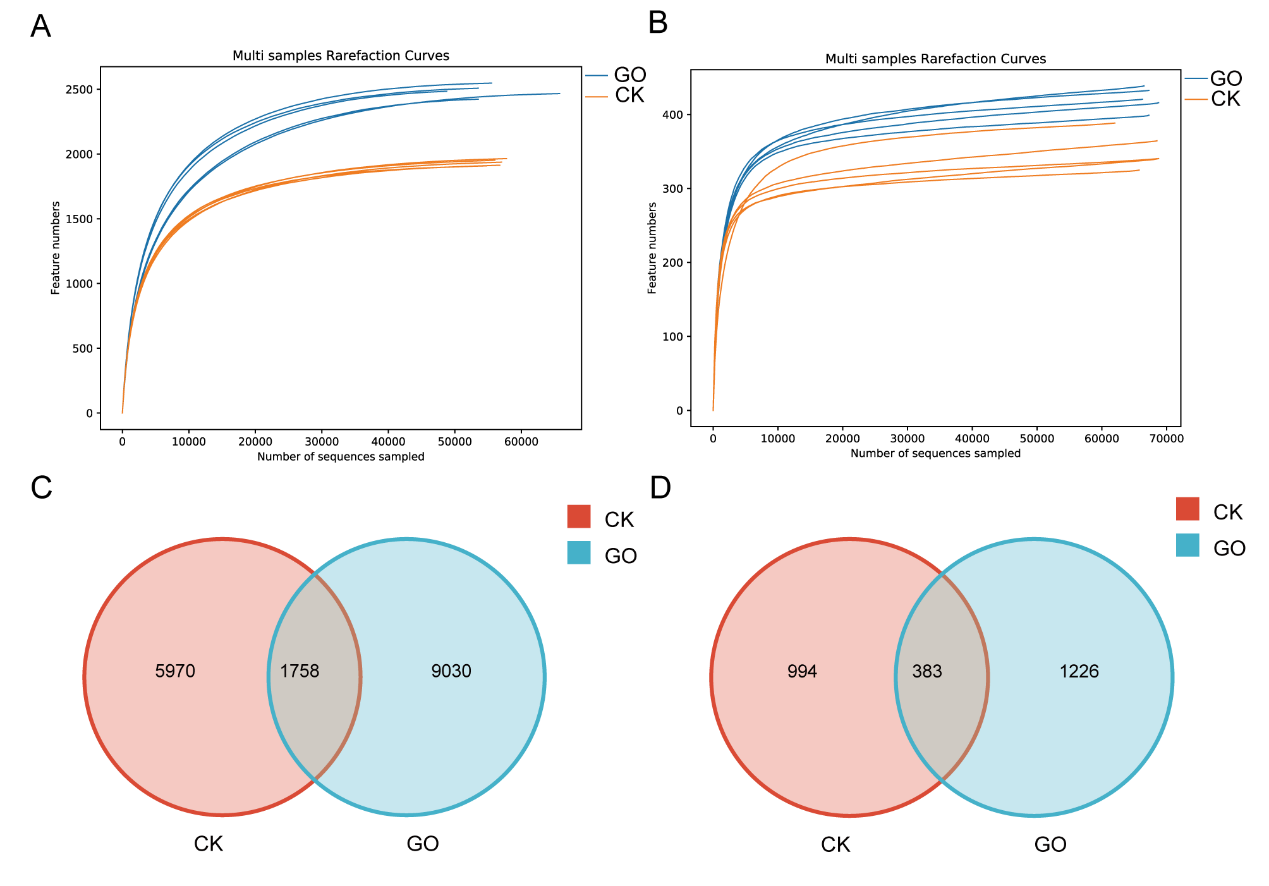


**Supplementary Figure 2.** Rhizosphere microbial sequencing of soybean cultivar L2012-7. **(A)** Rarefaction curve of bacteria based on OTU levels; **(B)** Rarefaction curve of fungi based on OTU levels. Venn diagrams of **(C)** bacterial OTU quantity and **(D)** fungal OTU quantity.


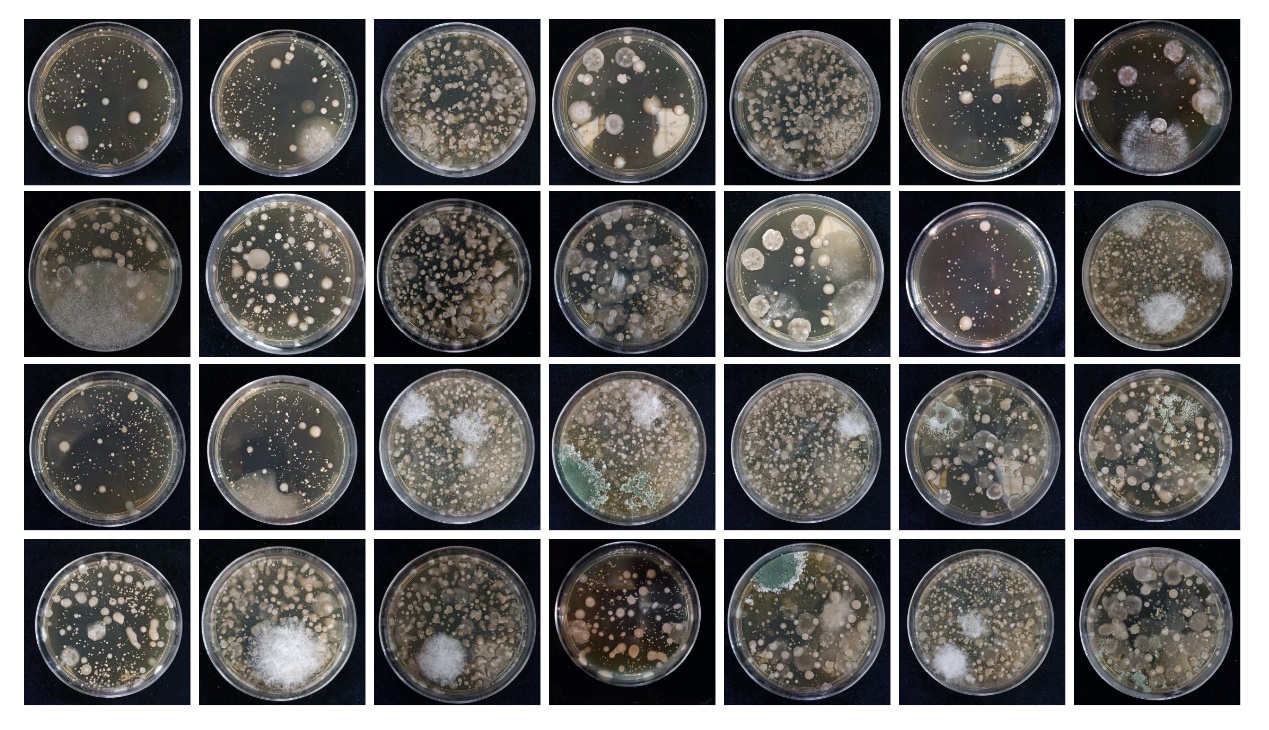


**Supplementary Figure 3.** Phenotypic images of some rhizosphere soil microorganisms from one soyabean cultivars L2012-7.


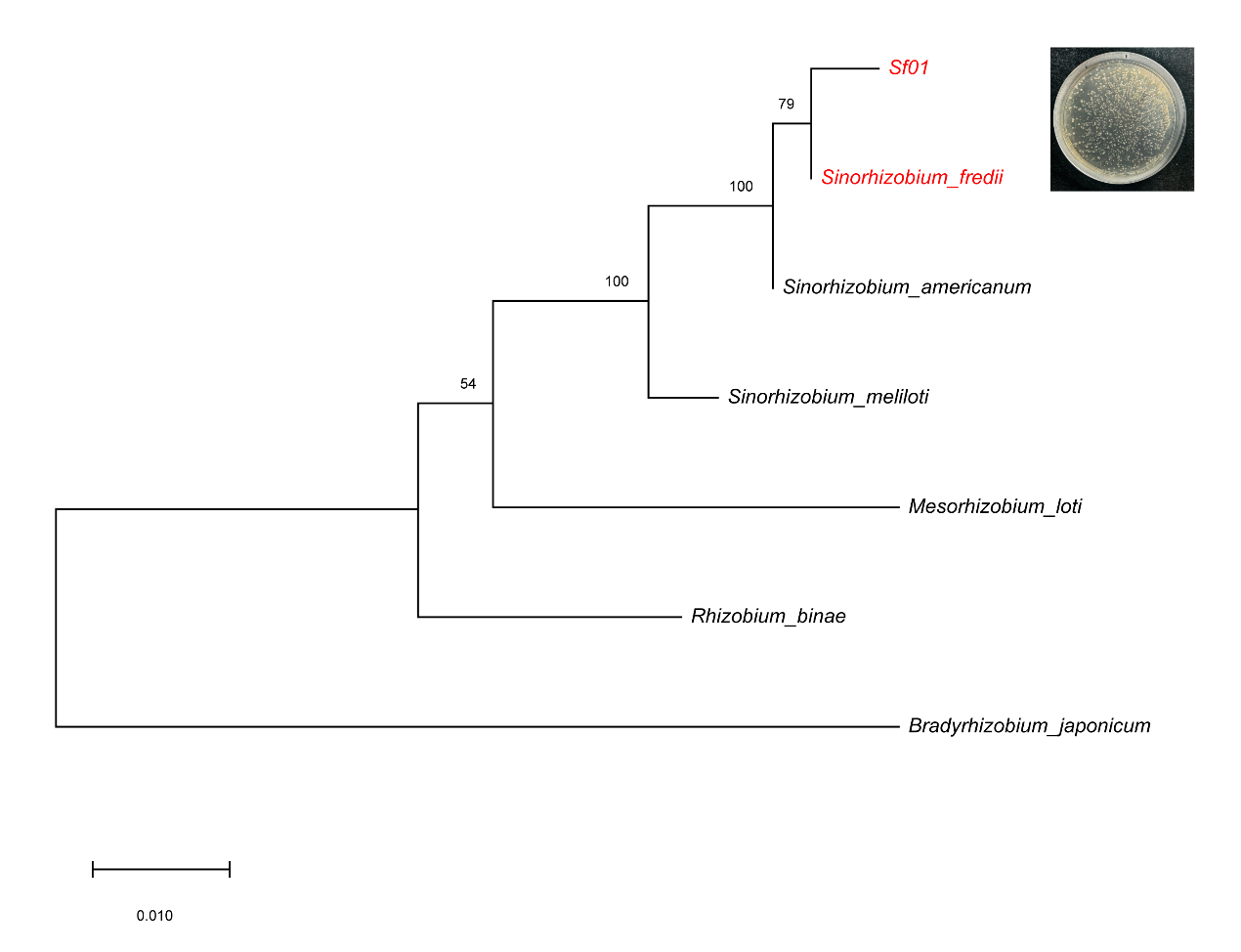


**Supplementary Figure 4.** Phylogenetic analysis of *Sf01* and others *rhizobia*. MEGA7 were used for generating the phylogenetic tree. The phylogenetic tree was inferred by the neighbour-joining algorithm. Rhizobium sequences are obtained from NCBI. Accession numbers are as follows: *Sinorhizobium fredii*, AB195268.1; *Sinorhizobium americanum*, CP013107.1; *Sinorhizobium meliloti*, NR_043399.1; *Mesorhizobium loti*, NZ_QGGH01000001.1; *Rhizobium binae*, NZ_CP071604.1; *Bradyrhizobium japonicum*, NZ_CP058354.1.


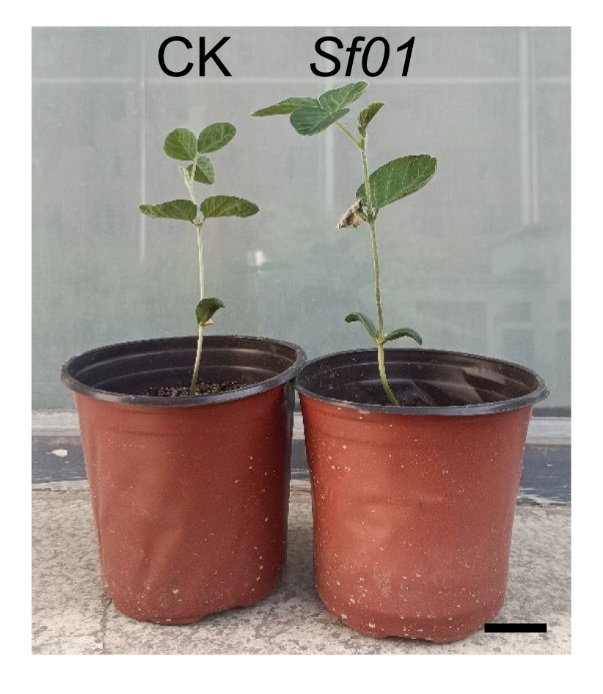


**Supplementary Figure 5.** *Sf01* promotes the growth of L2012-7 soybeans. The picture was taken one month after being treated with GO with a scale bar of 5 cm. CK refers to the control treatment with sterile water.


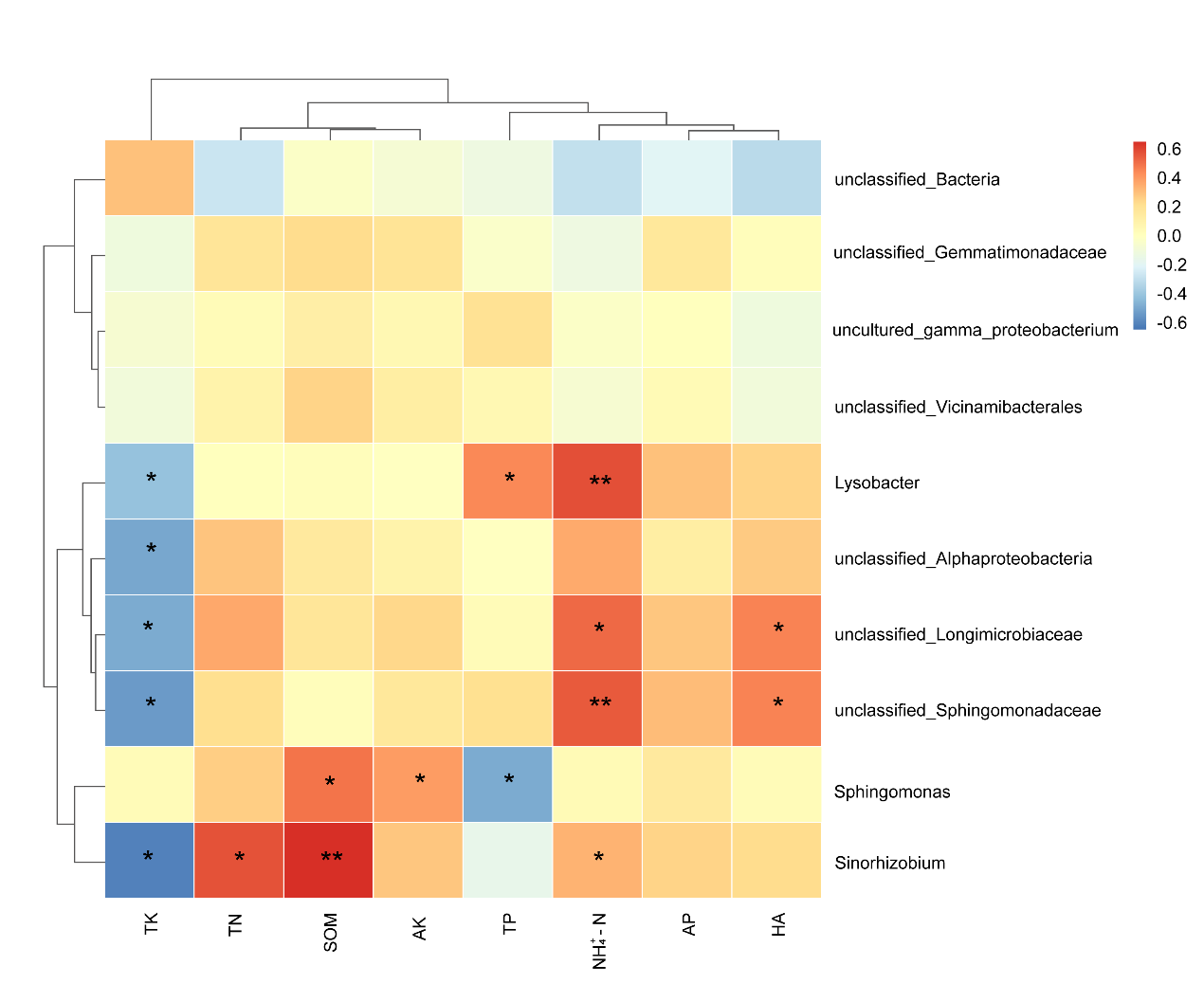


**Supplementary Figure 6.** Correlation analysis between key rhizosphere bacteria and soil nutrient content in soybeans. TK (total potassium), TN (total nitrogen), SOM (soil organic matter), AK (available potassium) , TP (total phosphorus), NH₄⁺-N (ammonium nitrogen), AP (available phosphorus) ,HA (humic acid).
